# Supplementary material for: Security awareness of single sign-on account in the academic community: the roles of demographics, privacy concerns, and Big-Five personality
Source: PeerJ Comput Sci. 2022 Mar 11;8:e918. doi: 10.7717/peerj-cs.918 (PMC9044249; doi:10.7717/peerj-cs.918)
Supplement: Supplemental Information 1 [file peerj-cs-08-918-s001.zip › SSOSurveyStudy.html]

SSOSurveyStudy


# SSOSurveyStudy

#### Ahmad R Pratama

#### 7/30/2021

# Preparation

## Packages

```
library(tidyverse)
library(summarytools)
library(ggfortify)
library(lm.beta)
library(sjPlot)
library(gridExtra)
library(dplyr)
library(hrbrthemes)
```

## Attach dataset

```
data <- read_csv("ssosurvey283.csv")
summary(data)
```

```
##        id           gender               age           roles          
##  Min.   :  1.0   Length:283         Min.   :17.00   Length:283        
##  1st Qu.: 71.5   Class :character   1st Qu.:19.00   Class :character  
##  Median :142.0   Mode  :character   Median :22.00   Mode  :character  
##  Mean   :142.0                      Mean   :26.63                     
##  3rd Qu.:212.5                      3rd Qu.:31.00                     
##  Max.   :283.0                      Max.   :59.00                     
##      score          knowledge         attitude         behavior     
##  Min.   : 34.50   Min.   : 25.00   Min.   : 15.00   Min.   : 25.00  
##  1st Qu.: 60.00   1st Qu.: 55.00   1st Qu.: 50.00   1st Qu.: 65.00  
##  Median : 67.50   Median : 65.00   Median : 60.00   Median : 75.00  
##  Mean   : 69.31   Mean   : 66.91   Mean   : 62.69   Mean   : 73.41  
##  3rd Qu.: 78.50   3rd Qu.: 80.00   3rd Qu.: 75.00   3rd Qu.: 85.00  
##  Max.   :100.00   Max.   :100.00   Max.   :100.00   Max.   :100.00  
##   familiarity        privacy       extraversion   agreeableness  
##  Min.   : 25.00   Min.   : 30.0   Min.   :1.000   Min.   :1.000  
##  1st Qu.: 75.00   1st Qu.: 80.0   1st Qu.:3.500   1st Qu.:4.500  
##  Median : 83.33   Median : 90.0   Median :4.000   Median :5.500  
##  Mean   : 80.86   Mean   : 85.9   Mean   :4.141   Mean   :5.302  
##  3rd Qu.:100.00   3rd Qu.:100.0   3rd Qu.:5.000   3rd Qu.:6.000  
##  Max.   :100.00   Max.   :100.0   Max.   :7.000   Max.   :7.000  
##  conscientiousness emotionalstability    openness           f1       
##  Min.   :2.500     Min.   :2.000      Min.   :1.500   Min.   :  0.0  
##  1st Qu.:4.500     1st Qu.:4.000      1st Qu.:4.500   1st Qu.: 75.0  
##  Median :5.000     Median :4.500      Median :5.500   Median : 75.0  
##  Mean   :5.138     Mean   :4.714      Mean   :5.327   Mean   : 82.6  
##  3rd Qu.:6.000     3rd Qu.:5.500      3rd Qu.:6.000   3rd Qu.:100.0  
##  Max.   :7.000     Max.   :7.000      Max.   :7.000   Max.   :100.0  
##        f2               f3              pr1              pr2        
##  Min.   :  0.00   Min.   :  0.00   Min.   :  0.00   Min.   :  0.00  
##  1st Qu.: 75.00   1st Qu.: 75.00   1st Qu.: 75.00   1st Qu.: 75.00  
##  Median : 75.00   Median :100.00   Median : 75.00   Median :100.00  
##  Mean   : 77.12   Mean   : 82.86   Mean   : 79.95   Mean   : 84.72  
##  3rd Qu.:100.00   3rd Qu.:100.00   3rd Qu.:100.00   3rd Qu.:100.00  
##  Max.   :100.00   Max.   :100.00   Max.   :100.00   Max.   :100.00  
##       pr3              pr4              pr5               k1     
##  Min.   :  0.00   Min.   :  0.00   Min.   :  0.00   Min.   :  0  
##  1st Qu.: 75.00   1st Qu.:100.00   1st Qu.: 75.00   1st Qu.: 25  
##  Median :100.00   Median :100.00   Median :100.00   Median : 50  
##  Mean   : 84.28   Mean   : 93.11   Mean   : 87.46   Mean   : 47  
##  3rd Qu.:100.00   3rd Qu.:100.00   3rd Qu.:100.00   3rd Qu.: 75  
##  Max.   :100.00   Max.   :100.00   Max.   :100.00   Max.   :100  
##        k2               k3               k4               k5        
##  Min.   :  0.00   Min.   :  0.00   Min.   :  0.00   Min.   :  0.00  
##  1st Qu.: 75.00   1st Qu.: 75.00   1st Qu.: 25.00   1st Qu.: 50.00  
##  Median :100.00   Median :100.00   Median : 50.00   Median : 75.00  
##  Mean   : 82.86   Mean   : 84.28   Mean   : 46.38   Mean   : 74.03  
##  3rd Qu.:100.00   3rd Qu.:100.00   3rd Qu.: 75.00   3rd Qu.:100.00  
##  Max.   :100.00   Max.   :100.00   Max.   :100.00   Max.   :100.00  
##        a1              a2               a3               a4        
##  Min.   :  0.0   Min.   :  0.00   Min.   :  0.00   Min.   :  0.00  
##  1st Qu.: 25.0   1st Qu.: 75.00   1st Qu.: 50.00   1st Qu.: 25.00  
##  Median : 50.0   Median :100.00   Median : 50.00   Median : 50.00  
##  Mean   : 51.5   Mean   : 80.83   Mean   : 60.51   Mean   : 42.84  
##  3rd Qu.: 75.0   3rd Qu.:100.00   3rd Qu.: 75.00   3rd Qu.: 75.00  
##  Max.   :100.0   Max.   :100.00   Max.   :100.00   Max.   :100.00  
##        a5               b1               b2               b3        
##  Min.   :  0.00   Min.   :  0.00   Min.   :  0.00   Min.   :  0.00  
##  1st Qu.: 50.00   1st Qu.: 75.00   1st Qu.: 75.00   1st Qu.: 75.00  
##  Median : 75.00   Median : 75.00   Median :100.00   Median : 75.00  
##  Mean   : 77.74   Mean   : 77.56   Mean   : 86.31   Mean   : 78.45  
##  3rd Qu.:100.00   3rd Qu.:100.00   3rd Qu.:100.00   3rd Qu.:100.00  
##  Max.   :100.00   Max.   :100.00   Max.   :100.00   Max.   :100.00  
##        b4            b5        
##  Min.   :  0   Min.   :  0.00  
##  1st Qu.: 50   1st Qu.: 25.00  
##  Median : 75   Median : 50.00  
##  Mean   : 75   Mean   : 49.73  
##  3rd Qu.:100   3rd Qu.: 75.00  
##  Max.   :100   Max.   :100.00
```

## Summary Statistics

```
data
```

```
## Warning: `...` is not empty.
## 
## We detected these problematic arguments:
## * `needs_dots`
## 
## These dots only exist to allow future extensions and should be empty.
## Did you misspecify an argument?
```

```
## # A tibble: 283 x 38
##       id gender   age roles score knowledge attitude behavior familiarity
##    <dbl> <chr>  <dbl> <chr> <dbl>     <dbl>    <dbl>    <dbl>       <dbl>
##  1     1 female    43 facu~  66.5        75       45       70        75  
##  2     2 female    48 facu~  88.5        90       95       85        83.3
##  3     3 male      41 staff  53          45       35       65        75  
##  4     4 male      45 staff  88.5        80       85       95       100  
##  5     5 male      47 staff  53          60       50       50       100  
##  6     6 female    51 facu~  74         100       45       70       100  
##  7     7 male      41 staff  63          65       55       65        75  
##  8     8 male      43 facu~  78.5        75       80       80        83.3
##  9     9 male      45 staff  82.5        90       65       85       100  
## 10    10 male      39 staff 100         100      100      100       100  
## # ... with 273 more rows, and 29 more variables: privacy <dbl>,
## #   extraversion <dbl>, agreeableness <dbl>, conscientiousness <dbl>,
## #   emotionalstability <dbl>, openness <dbl>, f1 <dbl>, f2 <dbl>, f3 <dbl>,
## #   pr1 <dbl>, pr2 <dbl>, pr3 <dbl>, pr4 <dbl>, pr5 <dbl>, k1 <dbl>, k2 <dbl>,
## #   k3 <dbl>, k4 <dbl>, k5 <dbl>, a1 <dbl>, a2 <dbl>, a3 <dbl>, a4 <dbl>,
## #   a5 <dbl>, b1 <dbl>, b2 <dbl>, b3 <dbl>, b4 <dbl>, b5 <dbl>
```

```
freq(data, report.nas = F)
```

```
## Variable(s) ignored: id, age, score
```

```
## Frequencies  
## data$gender  
## Type: Character  
## 
##                Freq        %   % Cum.
## ------------ ------ -------- --------
##       female    135    47.70    47.70
##         male    148    52.30   100.00
##        Total    283   100.00   100.00
## 
## data$roles  
## Type: Character  
## 
##                 Freq        %   % Cum.
## ------------- ------ -------- --------
##       faculty     34    12.01    12.01
##         staff     52    18.37    30.39
##       student    197    69.61   100.00
##         Total    283   100.00   100.00
## 
## data$knowledge  
## Type: Numeric  
## 
##               Freq        %   % Cum.
## ----------- ------ -------- --------
##          25      2     0.71     0.71
##          30      2     0.71     1.41
##          35      5     1.77     3.18
##          40      8     2.83     6.01
##          45     14     4.95    10.95
##          50     28     9.89    20.85
##          55     23     8.13    28.98
##          60     32    11.31    40.28
##          65     31    10.95    51.24
##          70     35    12.37    63.60
##          75     29    10.25    73.85
##          80     28     9.89    83.75
##          85     14     4.95    88.69
##          90     13     4.59    93.29
##          95      5     1.77    95.05
##         100     14     4.95   100.00
##       Total    283   100.00   100.00
## 
## data$attitude  
## Type: Numeric  
## 
##               Freq        %   % Cum.
## ----------- ------ -------- --------
##          15      1     0.35     0.35
##          20      6     2.12     2.47
##          25      1     0.35     2.83
##          30      5     1.77     4.59
##          35     10     3.53     8.13
##          40     18     6.36    14.49
##          45     16     5.65    20.14
##          50     31    10.95    31.10
##          55     30    10.60    41.70
##          60     31    10.95    52.65
##          65     27     9.54    62.19
##          70     19     6.71    68.90
##          75     19     6.71    75.62
##          80     22     7.77    83.39
##          85     16     5.65    89.05
##          90     10     3.53    92.58
##          95     10     3.53    96.11
##         100     11     3.89   100.00
##       Total    283   100.00   100.00
## 
## data$behavior  
## Type: Numeric  
## 
##               Freq        %   % Cum.
## ----------- ------ -------- --------
##          25      1     0.35     0.35
##          30      1     0.35     0.71
##          35      1     0.35     1.06
##          40      3     1.06     2.12
##          45      4     1.41     3.53
##          50     15     5.30     8.83
##          55     19     6.71    15.55
##          60     21     7.42    22.97
##          65     32    11.31    34.28
##          70     37    13.07    47.35
##          75     32    11.31    58.66
##          80     35    12.37    71.02
##          85     34    12.01    83.04
##          90     13     4.59    87.63
##          95     20     7.07    94.70
##         100     15     5.30   100.00
##       Total    283   100.00   100.00
## 
## data$familiarity  
## Type: Numeric  
## 
##               Freq        %   % Cum.
## ----------- ------ -------- --------
##          25      4     1.41     1.41
##       33.33      7     2.47     3.89
##       41.67      8     2.83     6.71
##          50     10     3.53    10.25
##       58.33      9     3.18    13.43
##       66.67     32    11.31    24.73
##          75     41    14.49    39.22
##       83.33     58    20.49    59.72
##       91.67     30    10.60    70.32
##         100     84    29.68   100.00
##       Total    283   100.00   100.00
## 
## data$privacy  
## Type: Numeric  
## 
##               Freq        %   % Cum.
## ----------- ------ -------- --------
##          30      3     1.06     1.06
##          35      1     0.35     1.41
##          45      3     1.06     2.47
##          50      5     1.77     4.24
##          55      3     1.06     5.30
##          60      4     1.41     6.71
##          65      9     3.18     9.89
##          70     14     4.95    14.84
##          75     25     8.83    23.67
##          80     29    10.25    33.92
##          85     33    11.66    45.58
##          90     36    12.72    58.30
##          95     42    14.84    73.14
##         100     76    26.86   100.00
##       Total    283   100.00   100.00
## 
## data$extraversion  
## Type: Numeric  
## 
##               Freq        %   % Cum.
## ----------- ------ -------- --------
##           1      1     0.35     0.35
##         1.5      7     2.47     2.83
##           2      7     2.47     5.30
##         2.5     16     5.65    10.95
##           3     31    10.95    21.91
##         3.5     41    14.49    36.40
##           4     51    18.02    54.42
##         4.5     47    16.61    71.02
##           5     33    11.66    82.69
##         5.5     29    10.25    92.93
##           6      8     2.83    95.76
##         6.5      4     1.41    97.17
##           7      8     2.83   100.00
##       Total    283   100.00   100.00
## 
## data$agreeableness  
## Type: Numeric  
## 
##               Freq        %   % Cum.
## ----------- ------ -------- --------
##           1      1     0.35     0.35
##         1.5      1     0.35     0.71
##           2      1     0.35     1.06
##         2.5      2     0.71     1.77
##           3      3     1.06     2.83
##         3.5      8     2.83     5.65
##           4     22     7.77    13.43
##         4.5     44    15.55    28.98
##           5     48    16.96    45.94
##         5.5     51    18.02    63.96
##           6     52    18.37    82.33
##         6.5     29    10.25    92.58
##           7     21     7.42   100.00
##       Total    283   100.00   100.00
## 
## data$conscientiousness  
## Type: Numeric  
## 
##               Freq        %   % Cum.
## ----------- ------ -------- --------
##         2.5      3     1.06     1.06
##           3     11     3.89     4.95
##         3.5     12     4.24     9.19
##           4     31    10.95    20.14
##         4.5     42    14.84    34.98
##           5     56    19.79    54.77
##         5.5     38    13.43    68.20
##           6     49    17.31    85.51
##         6.5     23     8.13    93.64
##           7     18     6.36   100.00
##       Total    283   100.00   100.00
## 
## data$emotionalstability  
## Type: Numeric  
## 
##               Freq        %   % Cum.
## ----------- ------ -------- --------
##           2      3     1.06     1.06
##         2.5     13     4.59     5.65
##           3     12     4.24     9.89
##         3.5     32    11.31    21.20
##           4     52    18.37    39.58
##         4.5     37    13.07    52.65
##           5     41    14.49    67.14
##         5.5     27     9.54    76.68
##           6     32    11.31    87.99
##         6.5     21     7.42    95.41
##           7     13     4.59   100.00
##       Total    283   100.00   100.00
## 
## data$openness  
## Type: Numeric  
## 
##               Freq        %   % Cum.
## ----------- ------ -------- --------
##         1.5      1     0.35     0.35
##           2      1     0.35     0.71
##         2.5      3     1.06     1.77
##           3      4     1.41     3.18
##         3.5      9     3.18     6.36
##           4     32    11.31    17.67
##         4.5     33    11.66    29.33
##           5     39    13.78    43.11
##         5.5     51    18.02    61.13
##           6     54    19.08    80.21
##         6.5     30    10.60    90.81
##           7     26     9.19   100.00
##       Total    283   100.00   100.00
## 
## data$f1  
## Type: Numeric  
## 
##               Freq        %   % Cum.
## ----------- ------ -------- --------
##           0      1     0.35     0.35
##          25      6     2.12     2.47
##          50     38    13.43    15.90
##          75     99    34.98    50.88
##         100    139    49.12   100.00
##       Total    283   100.00   100.00
## 
## data$f2  
## Type: Numeric  
## 
##               Freq        %   % Cum.
## ----------- ------ -------- --------
##           0      2     0.71     0.71
##          25     20     7.07     7.77
##          50     37    13.07    20.85
##          75    117    41.34    62.19
##         100    107    37.81   100.00
##       Total    283   100.00   100.00
## 
## data$f3  
## Type: Numeric  
## 
##               Freq        %   % Cum.
## ----------- ------ -------- --------
##           0      3     1.06     1.06
##          25     15     5.30     6.36
##          50     30    10.60    16.96
##          75     77    27.21    44.17
##         100    158    55.83   100.00
##       Total    283   100.00   100.00
## 
## data$pr1  
## Type: Numeric  
## 
##               Freq        %   % Cum.
## ----------- ------ -------- --------
##           0      1     0.35     0.35
##          25     10     3.53     3.89
##          50     36    12.72    16.61
##          75    121    42.76    59.36
##         100    115    40.64   100.00
##       Total    283   100.00   100.00
## 
## data$pr2  
## Type: Numeric  
## 
##               Freq        %   % Cum.
## ----------- ------ -------- --------
##           0      2     0.71     0.71
##          25      7     2.47     3.18
##          50     29    10.25    13.43
##          75     86    30.39    43.82
##         100    159    56.18   100.00
##       Total    283   100.00   100.00
## 
## data$pr3  
## Type: Numeric  
## 
##               Freq        %   % Cum.
## ----------- ------ -------- --------
##           0      3     1.06     1.06
##          25      6     2.12     3.18
##          50     23     8.13    11.31
##          75    102    36.04    47.35
##         100    149    52.65   100.00
##       Total    283   100.00   100.00
## 
## data$pr4  
## Type: Numeric  
## 
##               Freq        %   % Cum.
## ----------- ------ -------- --------
##           0      1     0.35     0.35
##          25      2     0.71     1.06
##          50      9     3.18     4.24
##          75     50    17.67    21.91
##         100    221    78.09   100.00
##       Total    283   100.00   100.00
## 
## data$pr5  
## Type: Numeric  
## 
##               Freq        %   % Cum.
## ----------- ------ -------- --------
##           0      1     0.35     0.35
##          25      8     2.83     3.18
##          50     21     7.42    10.60
##          75     72    25.44    36.04
##         100    181    63.96   100.00
##       Total    283   100.00   100.00
## 
## data$k1  
## Type: Numeric  
## 
##               Freq        %   % Cum.
## ----------- ------ -------- --------
##           0     57    20.14    20.14
##          25     66    23.32    43.46
##          50     66    23.32    66.78
##          75     42    14.84    81.63
##         100     52    18.37   100.00
##       Total    283   100.00   100.00
## 
## data$k2  
## Type: Numeric  
## 
##               Freq        %   % Cum.
## ----------- ------ -------- --------
##           0      9     3.18     3.18
##          25     12     4.24     7.42
##          50     32    11.31    18.73
##          75     58    20.49    39.22
##         100    172    60.78   100.00
##       Total    283   100.00   100.00
## 
## data$k3  
## Type: Numeric  
## 
##               Freq        %   % Cum.
## ----------- ------ -------- --------
##           0      8     2.83     2.83
##          25     12     4.24     7.07
##          50     26     9.19    16.25
##          75     58    20.49    36.75
##         100    179    63.25   100.00
##       Total    283   100.00   100.00
## 
## data$k4  
## Type: Numeric  
## 
##               Freq        %   % Cum.
## ----------- ------ -------- --------
##           0     55    19.43    19.43
##          25     67    23.67    43.11
##          50     67    23.67    66.78
##          75     52    18.37    85.16
##         100     42    14.84   100.00
##       Total    283   100.00   100.00
## 
## data$k5  
## Type: Numeric  
## 
##               Freq        %   % Cum.
## ----------- ------ -------- --------
##           0      8     2.83     2.83
##          25     18     6.36     9.19
##          50     57    20.14    29.33
##          75     94    33.22    62.54
##         100    106    37.46   100.00
##       Total    283   100.00   100.00
## 
## data$a1  
## Type: Numeric  
## 
##               Freq        %   % Cum.
## ----------- ------ -------- --------
##           0     40    14.13    14.13
##          25     66    23.32    37.46
##          50     69    24.38    61.84
##          75     53    18.73    80.57
##         100     55    19.43   100.00
##       Total    283   100.00   100.00
## 
## data$a2  
## Type: Numeric  
## 
##               Freq        %   % Cum.
## ----------- ------ -------- --------
##           0     16     5.65     5.65
##          25     14     4.95    10.60
##          50     26     9.19    19.79
##          75     59    20.85    40.64
##         100    168    59.36   100.00
##       Total    283   100.00   100.00
## 
## data$a3  
## Type: Numeric  
## 
##               Freq        %   % Cum.
## ----------- ------ -------- --------
##           0     22     7.77     7.77
##          25     41    14.49    22.26
##          50     79    27.92    50.18
##          75     78    27.56    77.74
##         100     63    22.26   100.00
##       Total    283   100.00   100.00
## 
## data$a4  
## Type: Numeric  
## 
##               Freq        %   % Cum.
## ----------- ------ -------- --------
##           0     51    18.02    18.02
##          25     90    31.80    49.82
##          50     61    21.55    71.38
##          75     51    18.02    89.40
##         100     30    10.60   100.00
##       Total    283   100.00   100.00
## 
## data$a5  
## Type: Numeric  
## 
##               Freq        %   % Cum.
## ----------- ------ -------- --------
##           0      4     1.41     1.41
##          25     14     4.95     6.36
##          50     54    19.08    25.44
##          75     86    30.39    55.83
##         100    125    44.17   100.00
##       Total    283   100.00   100.00
## 
## data$b1  
## Type: Numeric  
## 
##               Freq        %   % Cum.
## ----------- ------ -------- --------
##           0     10     3.53     3.53
##          25     18     6.36     9.89
##          50     42    14.84    24.73
##          75     76    26.86    51.59
##         100    137    48.41   100.00
##       Total    283   100.00   100.00
## 
## data$b2  
## Type: Numeric  
## 
##               Freq        %   % Cum.
## ----------- ------ -------- --------
##           0      5     1.77     1.77
##          25     11     3.89     5.65
##          50     26     9.19    14.84
##          75     50    17.67    32.51
##         100    191    67.49   100.00
##       Total    283   100.00   100.00
## 
## data$b3  
## Type: Numeric  
## 
##               Freq        %   % Cum.
## ----------- ------ -------- --------
##           0      7     2.47     2.47
##          25     14     4.95     7.42
##          50     37    13.07    20.49
##          75    100    35.34    55.83
##         100    125    44.17   100.00
##       Total    283   100.00   100.00
## 
## data$b4  
## Type: Numeric  
## 
##               Freq        %   % Cum.
## ----------- ------ -------- --------
##           0      8     2.83     2.83
##          25     17     6.01     8.83
##          50     59    20.85    29.68
##          75     82    28.98    58.66
##         100    117    41.34   100.00
##       Total    283   100.00   100.00
## 
## data$b5  
## Type: Numeric  
## 
##               Freq        %   % Cum.
## ----------- ------ -------- --------
##           0     38    13.43    13.43
##          25     66    23.32    36.75
##          50     86    30.39    67.14
##          75     47    16.61    83.75
##         100     46    16.25   100.00
##       Total    283   100.00   100.00
```

### Dependent Variables

```
dv <- data[, c('knowledge','k1','k2','k3','k4','k5','attitude','a1','a2','a3','a4','a5','behavior','b1','b2','b3','b4','b5','score')]
msd.dv <- dv %>% summarise_each(funs(mean, sd))
```

```
## Warning: `summarise_each_()` was deprecated in dplyr 0.7.0.
## Please use `across()` instead.
```

```
## Warning: `funs()` was deprecated in dplyr 0.8.0.
## Please use a list of either functions or lambdas: 
## 
##   # Simple named list: 
##   list(mean = mean, median = median)
## 
##   # Auto named with `tibble::lst()`: 
##   tibble::lst(mean, median)
## 
##   # Using lambdas
##   list(~ mean(., trim = .2), ~ median(., na.rm = TRUE))
```

```
round(msd.dv,digits=2)
```

```
## Warning: `...` is not empty.
## 
## We detected these problematic arguments:
## * `needs_dots`
## 
## These dots only exist to allow future extensions and should be empty.
## Did you misspecify an argument?
```

```
## # A tibble: 1 x 38
##   knowledge_mean k1_mean k2_mean k3_mean k4_mean k5_mean attitude_mean a1_mean
##            <dbl>   <dbl>   <dbl>   <dbl>   <dbl>   <dbl>         <dbl>   <dbl>
## 1           66.9      47    82.9    84.3    46.4    74.0          62.7    51.5
## # ... with 30 more variables: a2_mean <dbl>, a3_mean <dbl>, a4_mean <dbl>,
## #   a5_mean <dbl>, behavior_mean <dbl>, b1_mean <dbl>, b2_mean <dbl>,
## #   b3_mean <dbl>, b4_mean <dbl>, b5_mean <dbl>, score_mean <dbl>,
## #   knowledge_sd <dbl>, k1_sd <dbl>, k2_sd <dbl>, k3_sd <dbl>, k4_sd <dbl>,
## #   k5_sd <dbl>, attitude_sd <dbl>, a1_sd <dbl>, a2_sd <dbl>, a3_sd <dbl>,
## #   a4_sd <dbl>, a5_sd <dbl>, behavior_sd <dbl>, b1_sd <dbl>, b2_sd <dbl>,
## #   b3_sd <dbl>, b4_sd <dbl>, b5_sd <dbl>, score_sd <dbl>
```

### Independent Variables

```
iv <- data[, c('familiarity','f1','f2','f3','privacy','pr1','pr2','pr3','pr4','pr5','extraversion','agreeableness','conscientiousness','emotionalstability','openness')]
msd.iv <- iv %>% summarise_each(funs(mean, sd))
round(msd.iv,digits=2)
```

```
## Warning: `...` is not empty.
## 
## We detected these problematic arguments:
## * `needs_dots`
## 
## These dots only exist to allow future extensions and should be empty.
## Did you misspecify an argument?
```

```
## # A tibble: 1 x 30
##   familiarity_mean f1_mean f2_mean f3_mean privacy_mean pr1_mean pr2_mean
##              <dbl>   <dbl>   <dbl>   <dbl>        <dbl>    <dbl>    <dbl>
## 1             80.9    82.6    77.1    82.9         85.9     80.0     84.7
## # ... with 23 more variables: pr3_mean <dbl>, pr4_mean <dbl>, pr5_mean <dbl>,
## #   extraversion_mean <dbl>, agreeableness_mean <dbl>,
## #   conscientiousness_mean <dbl>, emotionalstability_mean <dbl>,
## #   openness_mean <dbl>, familiarity_sd <dbl>, f1_sd <dbl>, f2_sd <dbl>,
## #   f3_sd <dbl>, privacy_sd <dbl>, pr1_sd <dbl>, pr2_sd <dbl>, pr3_sd <dbl>,
## #   pr4_sd <dbl>, pr5_sd <dbl>, extraversion_sd <dbl>, agreeableness_sd <dbl>,
## #   conscientiousness_sd <dbl>, emotionalstability_sd <dbl>, openness_sd <dbl>
```

```
mean(data$age)
```

```
## [1] 26.62898
```

```
sd(data$age)
```

```
## [1] 10.22756
```

## Data Visualization

### Scatterplot Age - Score

```
g1 <- ggplot(data, aes(x=age, y=score, shape=gender, color=gender)) +
  geom_point() +
  geom_smooth(method=lm, se=FALSE, fullrange=TRUE)

g1 + facet_grid(rows = vars(roles))
```

```
## `geom_smooth()` using formula 'y ~ x'
```

```
g2 <- ggplot(data, aes(x=age, y=score, shape=roles, color=roles)) +
  geom_point() +
  geom_smooth(method=lm, se=FALSE, fullrange=TRUE)

g2 + facet_grid(rows = vars(gender))
```

```
## `geom_smooth()` using formula 'y ~ x'
```

```
g3 <- ggplot(data, aes(x=age, y=score, shape=gender, color=roles)) +
  geom_point() +
  geom_smooth(method=lm, se=FALSE, fullrange=TRUE)

g3 + facet_grid(vars(gender), vars(roles))+
   labs(title = "SSO Security Awareness by Age", x="Age (years old)", y="SSO Security Awareness Score")
```

```
## `geom_smooth()` using formula 'y ~ x'
```

```
#g3 + facet_grid(vars(roles), vars(gender))
```

### Score by Gender

```
scores <- data[,c('gender','score','knowledge','attitude','behavior')]
sg <- scores %>% 
    group_by(gender) %>%
      summarize_each(funs(mean))
sg
```

```
## Warning: `...` is not empty.
## 
## We detected these problematic arguments:
## * `needs_dots`
## 
## These dots only exist to allow future extensions and should be empty.
## Did you misspecify an argument?
```

```
## # A tibble: 2 x 5
##   gender score knowledge attitude behavior
##   <chr>  <dbl>     <dbl>    <dbl>    <dbl>
## 1 female  69.0      65.8     62.3     73.6
## 2 male    69.6      67.9     63.0     73.2
```

### Dumbbell Plots by Gender

```
tibble(
  Male = c(67.91, 63.04, 73.21, 69.59),
  Female = c(65.81, 62.30, 73.63, 69.02),
  cat = factor(c("Knowledge", "Attitude", "Behavior", "Total Score"), levels = c("Knowledge", "Attitude", "Behavior", "Total Score"))
) -> xdf

dp1 <- ggplot() +
  # reshape the data frame & get min value so you can draw an eye-tracking line (this is one geom)
  geom_segment(
    data = gather(xdf, measure, val, -cat) %>% 
      group_by(cat) %>% 
      top_n(-1) %>% 
      slice(1) %>%
      ungroup(),
    aes(x = 0, xend = val, y = cat, yend = cat),
    linetype = "dotted", size = 0.5, color = "gray80"
  ) +
  # reshape the data frame & get min/max category values so you can draw the segment (this is another geom)
  geom_segment(
    data = gather(xdf, measure, val, -cat) %>% 
      group_by(cat) %>% 
      summarise(start = range(val)[1], end = range(val)[2]) %>% 
      ungroup(),
    aes(x = start, xend = end, y = cat, yend = cat),
    color = "gray80", size = 1
  ) +
  # reshape the data frame & plot the points
  geom_point(
    data = gather(xdf, measure, value, -cat),
    aes(value, cat, color = measure), 
    size = 8
  ) +
  # i just extended the scale a bit + put axis on top; choose aesthetics that work 
  # for you
  scale_x_comma(position = "top", limits = c(50, 90)) +
  scale_color_ipsum(name = "Gender") +
  labs(
    x = NULL, y = NULL,
    title = "SSO Account Security Awareness By Gender"
  ) +
  theme_ipsum_rc(grid = "x") +
  theme(legend.position = "top")
```

```
## Selecting by val
```

```
dp1
```

```
## Warning: Removed 4 rows containing missing values (geom_segment).
```

```
## Warning in grid.Call(C_stringMetric, as.graphicsAnnot(x$label)): font family not
## found in Windows font database

## Warning in grid.Call(C_stringMetric, as.graphicsAnnot(x$label)): font family not
## found in Windows font database

## Warning in grid.Call(C_stringMetric, as.graphicsAnnot(x$label)): font family not
## found in Windows font database
```

```
## Warning in grid.Call(C_textBounds, as.graphicsAnnot(x$label), x$x, x$y, : font
## family not found in Windows font database

## Warning in grid.Call(C_textBounds, as.graphicsAnnot(x$label), x$x, x$y, : font
## family not found in Windows font database

## Warning in grid.Call(C_textBounds, as.graphicsAnnot(x$label), x$x, x$y, : font
## family not found in Windows font database

## Warning in grid.Call(C_textBounds, as.graphicsAnnot(x$label), x$x, x$y, : font
## family not found in Windows font database

## Warning in grid.Call(C_textBounds, as.graphicsAnnot(x$label), x$x, x$y, : font
## family not found in Windows font database

## Warning in grid.Call(C_textBounds, as.graphicsAnnot(x$label), x$x, x$y, : font
## family not found in Windows font database

## Warning in grid.Call(C_textBounds, as.graphicsAnnot(x$label), x$x, x$y, : font
## family not found in Windows font database
```

### Score by Roles

```
scores <- data[,c('roles','score','knowledge','attitude','behavior')]
sg <- scores %>% 
    group_by(roles) %>%
      summarize_each(funs(mean))
sg
```

```
## Warning: `...` is not empty.
## 
## We detected these problematic arguments:
## * `needs_dots`
## 
## These dots only exist to allow future extensions and should be empty.
## Did you misspecify an argument?
```

```
## # A tibble: 3 x 5
##   roles   score knowledge attitude behavior
##   <chr>   <dbl>     <dbl>    <dbl>    <dbl>
## 1 faculty  73.2      71.9     70.1     75.1
## 2 staff    71.2      70.5     63.6     74.7
## 3 student  68.1      65.1     61.2     72.8
```

### Dumbbell Plots by Roles

```
tibble(
  Student = c(65.0, 61.17, 72.77, 68.15),
  Faculty = c(71.91, 70.15, 75.15, 73.18),
  Staff = c(70.48, 63.56, 74.71, 71.21),
  cat = factor(c("Knowledge", "Attitude", "Behavior", "Total Score"), levels = c("Knowledge", "Attitude", "Behavior", "Total Score"))
) -> xdf

dp2 <- ggplot() +
  # reshape the data frame & get min value so you can draw an eye-tracking line (this is one geom)
  geom_segment(
    data = gather(xdf, measure, val, -cat) %>% 
      group_by(cat) %>% 
      top_n(-1) %>% 
      slice(1) %>%
      ungroup(),
    aes(x = 0, xend = val, y = cat, yend = cat),
    linetype = "dotted", size = 0.5, color = "gray80"
  ) +
  # reshape the data frame & get min/max category values so you can draw the segment (this is another geom)
  geom_segment(
    data = gather(xdf, measure, val, -cat) %>% 
      group_by(cat) %>% 
      summarise(start = range(val)[1], end = range(val)[2]) %>% 
      ungroup(),
    aes(x = start, xend = end, y = cat, yend = cat),
    color = "gray80", size = 1
  ) +
  # reshape the data frame & plot the points
  geom_point(
    data = gather(xdf, measure, value, -cat),
    aes(value, cat, color = measure), 
    size = 8
  ) +
  # i just extended the scale a bit + put axis on top; choose aesthetics that work 
  # for you
  scale_x_comma(position = "top", limits = c(50, 90)) +
  scale_color_ipsum(name = "Academic Role") +
  labs(
    x = NULL, y = NULL,
    title = "SSO Account Security Awareness By Role"
  ) +
  theme_ipsum_rc(grid = "x") +
  theme(legend.position = "top")
```

```
## Selecting by val
```

```
dp2
```

```
## Warning: Removed 4 rows containing missing values (geom_segment).
```

```
## Warning in grid.Call(C_textBounds, as.graphicsAnnot(x$label), x$x, x$y, : font
## family not found in Windows font database

## Warning in grid.Call(C_textBounds, as.graphicsAnnot(x$label), x$x, x$y, : font
## family not found in Windows font database

## Warning in grid.Call(C_textBounds, as.graphicsAnnot(x$label), x$x, x$y, : font
## family not found in Windows font database

## Warning in grid.Call(C_textBounds, as.graphicsAnnot(x$label), x$x, x$y, : font
## family not found in Windows font database

## Warning in grid.Call(C_textBounds, as.graphicsAnnot(x$label), x$x, x$y, : font
## family not found in Windows font database

## Warning in grid.Call(C_textBounds, as.graphicsAnnot(x$label), x$x, x$y, : font
## family not found in Windows font database

## Warning in grid.Call(C_textBounds, as.graphicsAnnot(x$label), x$x, x$y, : font
## family not found in Windows font database

## Warning in grid.Call(C_textBounds, as.graphicsAnnot(x$label), x$x, x$y, : font
## family not found in Windows font database

## Warning in grid.Call(C_textBounds, as.graphicsAnnot(x$label), x$x, x$y, : font
## family not found in Windows font database
```

## OLS Regression

### Model 1: Privacy

```
model1 <- lm(score ~ gender + age + roles + familiarity + privacy, data = data)
summary(model1)
```

```
## 
## Call:
## lm(formula = score ~ gender + age + roles + familiarity + privacy, 
##     data = data)
## 
## Residuals:
##     Min      1Q  Median      3Q     Max 
## -36.754  -8.548  -0.625   8.717  34.228 
## 
## Coefficients:
##               Estimate Std. Error t value Pr(>|t|)    
## (Intercept)   65.18460    8.84774   7.367 2.02e-12 ***
## gendermale     0.12303    1.58773   0.077 0.938293    
## age           -0.32118    0.14053  -2.285 0.023045 *  
## rolesstaff    -2.40222    2.88298  -0.833 0.405429    
## rolesstudent -12.81880    3.56549  -3.595 0.000384 ***
## familiarity    0.11657    0.04330   2.692 0.007529 ** 
## privacy        0.14618    0.05873   2.489 0.013405 *  
## ---
## Signif. codes:  0 '***' 0.001 '**' 0.01 '*' 0.05 '.' 0.1 ' ' 1
## 
## Residual standard error: 12.99 on 276 degrees of freedom
## Multiple R-squared:  0.1098, Adjusted R-squared:  0.09043 
## F-statistic: 5.673 on 6 and 276 DF,  p-value: 1.413e-05
```

### Model 2: Privacy + Big5

```
model2 <- lm(score ~ gender + age + roles + familiarity + privacy + extraversion + agreeableness + conscientiousness + emotionalstability + openness, data = data)
summary(model2)
```

```
## 
## Call:
## lm(formula = score ~ gender + age + roles + familiarity + privacy + 
##     extraversion + agreeableness + conscientiousness + emotionalstability + 
##     openness, data = data)
## 
## Residuals:
##     Min      1Q  Median      3Q     Max 
## -39.680  -8.343  -0.343   7.925  34.050 
## 
## Coefficients:
##                     Estimate Std. Error t value Pr(>|t|)    
## (Intercept)         67.23559    9.74161   6.902 3.63e-11 ***
## gendermale          -0.44824    1.60975  -0.278 0.780877    
## age                 -0.30369    0.14087  -2.156 0.031980 *  
## rolesstaff          -2.73726    2.86568  -0.955 0.340335    
## rolesstudent       -11.97743    3.54832  -3.376 0.000845 ***
## familiarity          0.09744    0.04514   2.159 0.031754 *  
## privacy              0.14568    0.05899   2.470 0.014139 *  
## extraversion        -1.20486    0.68055  -1.770 0.077781 .  
## agreeableness       -1.31153    0.86795  -1.511 0.131935    
## conscientiousness    1.67437    0.89535   1.870 0.062551 .  
## emotionalstability   1.17025    0.81947   1.428 0.154428    
## openness            -0.62474    0.85806  -0.728 0.467191    
## ---
## Signif. codes:  0 '***' 0.001 '**' 0.01 '*' 0.05 '.' 0.1 ' ' 1
## 
## Residual standard error: 12.84 on 271 degrees of freedom
## Multiple R-squared:  0.1464, Adjusted R-squared:  0.1118 
## F-statistic: 4.227 on 11 and 271 DF,  p-value: 8.502e-06
```

### Model 3: Privacy x Big5

```
model3 <- lm(score ~ gender + age + roles + familiarity + privacy + extraversion + agreeableness*privacy + conscientiousness*privacy + emotionalstability + openness, data = data)
summary(model3)
```

```
## 
## Call:
## lm(formula = score ~ gender + age + roles + familiarity + privacy + 
##     extraversion + agreeableness * privacy + conscientiousness * 
##     privacy + emotionalstability + openness, data = data)
## 
## Residuals:
##     Min      1Q  Median      3Q     Max 
## -38.631  -8.918  -0.500   8.273  33.953 
## 
## Coefficients:
##                            Estimate Std. Error t value Pr(>|t|)    
## (Intercept)                66.99399   26.48445   2.530 0.011992 *  
## gendermale                 -0.52353    1.58828  -0.330 0.741943    
## age                        -0.30919    0.13895  -2.225 0.026894 *  
## rolesstaff                 -2.85076    2.82864  -1.008 0.314446    
## rolesstudent              -12.36247    3.50555  -3.527 0.000495 ***
## familiarity                 0.09727    0.04478   2.172 0.030714 *  
## privacy                     0.15591    0.29570   0.527 0.598459    
## extraversion               -1.04614    0.67326  -1.554 0.121398    
## agreeableness             -16.28630    5.22743  -3.116 0.002035 ** 
## conscientiousness          16.07013    5.59876   2.870 0.004427 ** 
## emotionalstability          1.43220    0.81263   1.762 0.079134 .  
## openness                   -0.62294    0.84691  -0.736 0.462648    
## privacy:agreeableness       0.16675    0.05781   2.884 0.004240 ** 
## privacy:conscientiousness  -0.16493    0.06394  -2.580 0.010422 *  
## ---
## Signif. codes:  0 '***' 0.001 '**' 0.01 '*' 0.05 '.' 0.1 ' ' 1
## 
## Residual standard error: 12.66 on 269 degrees of freedom
## Multiple R-squared:  0.176,  Adjusted R-squared:  0.1361 
## F-statistic: 4.418 on 13 and 269 DF,  p-value: 8.681e-07
```

## Diagnostics

### Residuals vs Fitted

```
plot(model1, 1)
```

```
plot(model2, 1)
```

```
plot(model3, 1)
```

### Normal Q-Q

```
plot(model1, 2)
```

```
plot(model2, 2)
```

```
plot(model3, 2)
```

### Scale-Location

```
plot(model1, 3)
```

```
plot(model2, 3)
```

```
plot(model3, 3)
```

### Cook’s distance

```
plot(model1, 4)
```

```
plot(model2, 4)
```

```
plot(model3, 4)
```

### Residuals vs Leverage

```
plot(model1, 5)
```

```
plot(model2, 5)
```

```
plot(model3, 5)
```

### Other Approaches

```
library("car")
```

```
## Loading required package: carData
```

```
## Registered S3 methods overwritten by 'car':
##   method                          from
##   influence.merMod                lme4
##   cooks.distance.influence.merMod lme4
##   dfbeta.influence.merMod         lme4
##   dfbetas.influence.merMod        lme4
```

```
## 
## Attaching package: 'car'
```

```
## The following object is masked from 'package:dplyr':
## 
##     recode
```

```
## The following object is masked from 'package:purrr':
## 
##     some
```

```
qqPlot(model2,labels=row.names(id), id.method="identify", simulate=TRUE, main="Q-Q Plot")
```

```
## [1] 128 252
```

```
qqPlot(model3,labels=row.names(id), id.method="identify", simulate=TRUE, main="Q-Q Plot")
```

```
## [1] 198 252
```

```
outlierTest(model2)
```

```
## No Studentized residuals with Bonferroni p < 0.05
## Largest |rstudent|:
##      rstudent unadjusted p-value Bonferroni p
## 252 -3.183166          0.0016272       0.4605
```

```
outlierTest(model3)
```

```
## No Studentized residuals with Bonferroni p < 0.05
## Largest |rstudent|:
##      rstudent unadjusted p-value Bonferroni p
## 252 -3.145981          0.0018419      0.52126
```

```
influencePlot(model2, main="Influence Plot", sub="Circle size is proportional to Cook’s distance")
```

```
##       StudRes        Hat      CookD
## 128  2.791278 0.07456718 0.05103623
## 246  2.374304 0.11493676 0.05998014
## 252 -3.183166 0.02550734 0.02138107
## 277 -1.305121 0.13296805 0.02171235
```

```
influencePlot(model3, main="Influence Plot", sub="Circle size is proportional to Cook’s distance")
```

```
##        StudRes        Hat      CookD
## 16  -0.9381115 0.30549679 0.02766349
## 66   0.9824222 0.24707779 0.02262602
## 128  2.3023026 0.10340082 0.04297681
## 198 -2.8471873 0.04038638 0.02374205
## 246  2.3840589 0.11580305 0.05226130
## 252 -3.1459809 0.02833914 0.01995833
```

```
qqPlot(model1,labels=row.names(id), id.method="identify", simulate=TRUE, main="Q-Q Plot")
```

```
## [1] 220 252
```

```
highleverage <- function(fit) {
 p <- length(coefficients(fit))
 n <- length(fitted(fit))
 ratio <-p/n
 plot(hatvalues(fit), main="Index Plot of Ratio")
 abline(h=c(2,3)*ratio, col="red", lty=2)
 text(hatvalues(fit), labels=rownames(data), font = 2)
}
highleverage(model1)
```

```
influencePlot(model1, main="Influence Plot", sub="Circle size is proportional to Cook’s distance")
```

```
##        StudRes        Hat        CookD
## 66   2.1124826 0.08481837 0.0583520737
## 68   0.1571987 0.09643844 0.0003781202
## 70  -0.3572603 0.09213112 0.0018562201
## 101  2.4419736 0.04011204 0.0349701631
## 220  2.6822861 0.01361101 0.0138712169
## 252 -2.8880125 0.01496857 0.0176372373
```

```
qqPlot(model3,labels=row.names(id), id.method="identify", simulate=TRUE, main="Q-Q Plot")
```

```
## [1] 198 252
```

```
highleverage <- function(fit) {
 p <- length(coefficients(fit))
 n <- length(fitted(fit))
 ratio <-p/n
 plot(hatvalues(fit), main="Index Plot of Ratio")
 abline(h=c(2,3)*ratio, col="red", lty=2)
 text(hatvalues(fit), labels=rownames(data), font = 2)
}
highleverage(model3)
```

```
influencePlot(model3, main="Influence Plot", sub="Circle size is proportional to Cook’s distance")
```

```
##        StudRes        Hat      CookD
## 16  -0.9381115 0.30549679 0.02766349
## 66   0.9824222 0.24707779 0.02262602
## 128  2.3023026 0.10340082 0.04297681
## 198 -2.8471873 0.04038638 0.02374205
## 246  2.3840589 0.11580305 0.05226130
## 252 -3.1459809 0.02833914 0.01995833
```

## Without Outliers, High-Leverage, & Influential Cases

```
data2 <- data[-c(101, 252, 169),]
summary(data2)
```

```
##        id            gender               age           roles          
##  Min.   :  1.00   Length:280         Min.   :17.00   Length:280        
##  1st Qu.: 70.75   Class :character   1st Qu.:19.00   Class :character  
##  Median :141.50   Mode  :character   Median :21.50   Mode  :character  
##  Mean   :141.66                      Mean   :26.63                     
##  3rd Qu.:212.25                      3rd Qu.:31.00                     
##  Max.   :283.00                      Max.   :59.00                     
##      score          knowledge        attitude        behavior     
##  Min.   : 39.50   Min.   : 25.0   Min.   : 15.0   Min.   : 30.00  
##  1st Qu.: 60.00   1st Qu.: 55.0   1st Qu.: 50.0   1st Qu.: 65.00  
##  Median : 67.50   Median : 65.0   Median : 60.0   Median : 75.00  
##  Mean   : 69.25   Mean   : 66.8   Mean   : 62.5   Mean   : 73.41  
##  3rd Qu.: 78.50   3rd Qu.: 80.0   3rd Qu.: 75.0   3rd Qu.: 85.00  
##  Max.   :100.00   Max.   :100.0   Max.   :100.0   Max.   :100.00  
##   familiarity        privacy        extraversion   agreeableness  
##  Min.   : 25.00   Min.   : 30.00   Min.   :1.000   Min.   :1.000  
##  1st Qu.: 72.92   1st Qu.: 80.00   1st Qu.:3.500   1st Qu.:4.500  
##  Median : 83.33   Median : 90.00   Median :4.000   Median :5.500  
##  Mean   : 80.71   Mean   : 85.86   Mean   :4.138   Mean   :5.318  
##  3rd Qu.:100.00   3rd Qu.:100.00   3rd Qu.:5.000   3rd Qu.:6.000  
##  Max.   :100.00   Max.   :100.00   Max.   :7.000   Max.   :7.000  
##  conscientiousness emotionalstability    openness           f1        
##  Min.   :2.500     Min.   :2.00       Min.   :1.500   Min.   :  0.00  
##  1st Qu.:4.500     1st Qu.:4.00       1st Qu.:4.500   1st Qu.: 75.00  
##  Median :5.000     Median :4.50       Median :5.500   Median : 75.00  
##  Mean   :5.132     Mean   :4.72       Mean   :5.316   Mean   : 82.41  
##  3rd Qu.:6.000     3rd Qu.:5.50       3rd Qu.:6.000   3rd Qu.:100.00  
##  Max.   :7.000     Max.   :7.00       Max.   :7.000   Max.   :100.00  
##        f2               f3              pr1              pr2        
##  Min.   :  0.00   Min.   :  0.00   Min.   :  0.00   Min.   :  0.00  
##  1st Qu.: 75.00   1st Qu.: 75.00   1st Qu.: 75.00   1st Qu.: 75.00  
##  Median : 75.00   Median :100.00   Median : 75.00   Median :100.00  
##  Mean   : 76.96   Mean   : 82.77   Mean   : 79.82   Mean   : 84.73  
##  3rd Qu.:100.00   3rd Qu.:100.00   3rd Qu.:100.00   3rd Qu.:100.00  
##  Max.   :100.00   Max.   :100.00   Max.   :100.00   Max.   :100.00  
##       pr3             pr4              pr5              k1        
##  Min.   :  0.0   Min.   :  0.00   Min.   :  0.0   Min.   :  0.00  
##  1st Qu.: 75.0   1st Qu.:100.00   1st Qu.: 75.0   1st Qu.: 25.00  
##  Median :100.0   Median :100.00   Median :100.0   Median : 50.00  
##  Mean   : 84.2   Mean   : 93.04   Mean   : 87.5   Mean   : 46.79  
##  3rd Qu.:100.0   3rd Qu.:100.00   3rd Qu.:100.0   3rd Qu.: 75.00  
##  Max.   :100.0   Max.   :100.00   Max.   :100.0   Max.   :100.00  
##        k2               k3               k4               k5        
##  Min.   :  0.00   Min.   :  0.00   Min.   :  0.00   Min.   :  0.00  
##  1st Qu.: 75.00   1st Qu.: 75.00   1st Qu.: 25.00   1st Qu.: 50.00  
##  Median :100.00   Median :100.00   Median : 50.00   Median : 75.00  
##  Mean   : 82.95   Mean   : 84.29   Mean   : 46.07   Mean   : 73.93  
##  3rd Qu.:100.00   3rd Qu.:100.00   3rd Qu.: 75.00   3rd Qu.:100.00  
##  Max.   :100.00   Max.   :100.00   Max.   :100.00   Max.   :100.00  
##        a1               a2               a3               a4        
##  Min.   :  0.00   Min.   :  0.00   Min.   :  0.00   Min.   :  0.00  
##  1st Qu.: 25.00   1st Qu.: 75.00   1st Qu.: 50.00   1st Qu.: 25.00  
##  Median : 50.00   Median :100.00   Median : 50.00   Median : 50.00  
##  Mean   : 51.07   Mean   : 80.71   Mean   : 60.27   Mean   : 42.77  
##  3rd Qu.: 75.00   3rd Qu.:100.00   3rd Qu.: 75.00   3rd Qu.: 75.00  
##  Max.   :100.00   Max.   :100.00   Max.   :100.00   Max.   :100.00  
##        a5               b1               b2               b3        
##  Min.   :  0.00   Min.   :  0.00   Min.   :  0.00   Min.   :  0.00  
##  1st Qu.: 50.00   1st Qu.: 68.75   1st Qu.: 75.00   1st Qu.: 75.00  
##  Median : 75.00   Median : 75.00   Median :100.00   Median : 75.00  
##  Mean   : 77.68   Mean   : 77.32   Mean   : 86.43   Mean   : 78.57  
##  3rd Qu.:100.00   3rd Qu.:100.00   3rd Qu.:100.00   3rd Qu.:100.00  
##  Max.   :100.00   Max.   :100.00   Max.   :100.00   Max.   :100.00  
##        b4               b5        
##  Min.   :  0.00   Min.   :  0.00  
##  1st Qu.: 50.00   1st Qu.: 25.00  
##  Median : 75.00   Median : 50.00  
##  Mean   : 75.09   Mean   : 49.64  
##  3rd Qu.:100.00   3rd Qu.: 75.00  
##  Max.   :100.00   Max.   :100.00
```

### Model 01: Demographics Only

```
model0b <- lm(score ~ gender + age + roles, data = data2)
summary(model0b)
```

```
## 
## Call:
## lm(formula = score ~ gender + age + roles, data = data2)
## 
## Residuals:
##     Min      1Q  Median      3Q     Max 
## -27.455  -9.518  -1.674   9.551  32.434 
## 
## Coefficients:
##              Estimate Std. Error t value Pr(>|t|)    
## (Intercept)   93.3716     6.0199  15.510  < 2e-16 ***
## gendermale     0.1243     1.5878   0.078 0.937677    
## age           -0.5034     0.1384  -3.638 0.000328 ***
## rolesstaff    -2.6556     2.8780  -0.923 0.356959    
## rolesstudent -14.8560     3.5990  -4.128 4.86e-05 ***
## ---
## Signif. codes:  0 '***' 0.001 '**' 0.01 '*' 0.05 '.' 0.1 ' ' 1
## 
## Residual standard error: 12.99 on 275 degrees of freedom
## Multiple R-squared:  0.06545,    Adjusted R-squared:  0.05186 
## F-statistic: 4.815 on 4 and 275 DF,  p-value: 0.0009075
```

### Model 02: Familiarity

```
model0c <- lm(score ~ gender + age + roles + familiarity, data = data2)
summary(model0c)
```

```
## 
## Call:
## lm(formula = score ~ gender + age + roles + familiarity, data = data2)
## 
## Residuals:
##     Min      1Q  Median      3Q     Max 
## -29.689  -8.448  -1.488   8.826  32.602 
## 
## Coefficients:
##               Estimate Std. Error t value Pr(>|t|)    
## (Intercept)   80.01852    7.14831  11.194  < 2e-16 ***
## gendermale    -0.32182    1.56535  -0.206  0.83726    
## age           -0.43465    0.13746  -3.162  0.00174 ** 
## rolesstaff    -2.80007    2.82714  -0.990  0.32284    
## rolesstudent -14.01176    3.54407  -3.954 9.81e-05 ***
## familiarity    0.13875    0.04174   3.324  0.00101 ** 
## ---
## Signif. codes:  0 '***' 0.001 '**' 0.01 '*' 0.05 '.' 0.1 ' ' 1
## 
## Residual standard error: 12.76 on 274 degrees of freedom
## Multiple R-squared:  0.1017, Adjusted R-squared:  0.08528 
## F-statistic: 6.203 on 5 and 274 DF,  p-value: 1.817e-05
```

### Model 1: Privacy

```
model1b <- lm(score ~ gender + age + roles + familiarity + privacy, data = data2)
summary(model1b)
```

```
## 
## Call:
## lm(formula = score ~ gender + age + roles + familiarity + privacy, 
##     data = data2)
## 
## Residuals:
##     Min      1Q  Median      3Q     Max 
## -31.016  -8.509  -0.411   8.484  34.556 
## 
## Coefficients:
##               Estimate Std. Error t value Pr(>|t|)    
## (Intercept)   66.36537    8.67561   7.650 3.47e-13 ***
## gendermale    -0.13503    1.54903  -0.087 0.930601    
## age           -0.36037    0.13862  -2.600 0.009841 ** 
## rolesstaff    -2.40412    2.79870  -0.859 0.391087    
## rolesstudent -13.79422    3.50456  -3.936 0.000105 ***
## familiarity    0.11473    0.04220   2.718 0.006980 ** 
## privacy        0.15481    0.05706   2.713 0.007093 ** 
## ---
## Signif. codes:  0 '***' 0.001 '**' 0.01 '*' 0.05 '.' 0.1 ' ' 1
## 
## Residual standard error: 12.61 on 273 degrees of freedom
## Multiple R-squared:  0.1253, Adjusted R-squared:  0.106 
## F-statistic: 6.515 on 6 and 273 DF,  p-value: 1.927e-06
```

```
lm.beta(model1b)
```

```
## 
## Call:
## lm(formula = score ~ gender + age + roles + familiarity + privacy, 
##     data = data2)
## 
## Standardized Coefficients::
##  (Intercept)   gendermale          age   rolesstaff rolesstudent  familiarity 
##   0.00000000  -0.00506419  -0.27685103  -0.07021533  -0.47791925   0.15988792 
##      privacy 
##   0.16934345
```

### Model 2: Privacy + Big5

```
model2b <- lm(score ~ gender + age + roles + familiarity + privacy + extraversion + agreeableness + conscientiousness + emotionalstability + openness, data = data2)
summary(model2b)
```

```
## 
## Call:
## lm(formula = score ~ gender + age + roles + familiarity + privacy + 
##     extraversion + agreeableness + conscientiousness + emotionalstability + 
##     openness, data = data2)
## 
## Residuals:
##     Min      1Q  Median      3Q     Max 
## -33.356  -8.588  -0.325   7.604  33.897 
## 
## Coefficients:
##                     Estimate Std. Error t value Pr(>|t|)    
## (Intercept)         67.83200    9.48756   7.150 8.28e-12 ***
## gendermale          -0.76677    1.56602  -0.490 0.624799    
## age                 -0.34416    0.13829  -2.489 0.013431 *  
## rolesstaff          -2.77278    2.76749  -1.002 0.317290    
## rolesstudent       -12.87717    3.46399  -3.717 0.000245 ***
## familiarity          0.09941    0.04363   2.279 0.023475 *  
## privacy              0.14847    0.05703   2.604 0.009739 ** 
## extraversion        -1.46799    0.65989  -2.225 0.026941 *  
## agreeableness       -0.92773    0.86110  -1.077 0.282283    
## conscientiousness    1.56834    0.87426   1.794 0.073955 .  
## emotionalstability   1.36944    0.79269   1.728 0.085217 .  
## openness            -0.72560    0.83334  -0.871 0.384691    
## ---
## Signif. codes:  0 '***' 0.001 '**' 0.01 '*' 0.05 '.' 0.1 ' ' 1
## 
## Residual standard error: 12.4 on 268 degrees of freedom
## Multiple R-squared:  0.1701, Adjusted R-squared:  0.136 
## F-statistic: 4.993 on 11 and 268 DF,  p-value: 4.518e-07
```

```
lm.beta(model2b)
```

```
## 
## Call:
## lm(formula = score ~ gender + age + roles + familiarity + privacy + 
##     extraversion + agreeableness + conscientiousness + emotionalstability + 
##     openness, data = data2)
## 
## Standardized Coefficients::
##        (Intercept)         gendermale                age         rolesstaff 
##         0.00000000        -0.02875771        -0.26440075        -0.08098268 
##       rolesstudent        familiarity            privacy       extraversion 
##        -0.44614668         0.13853703         0.16240736        -0.12969442 
##      agreeableness  conscientiousness emotionalstability           openness 
##        -0.07038329         0.12295752         0.12296731        -0.05837435
```

### Model 3: Privacy x Big5

```
model3b <- lm(score ~ gender + age + roles + familiarity + privacy + extraversion + agreeableness*privacy + conscientiousness*privacy + emotionalstability + openness, data = data2)
summary(model3b)
```

```
## 
## Call:
## lm(formula = score ~ gender + age + roles + familiarity + privacy + 
##     extraversion + agreeableness * privacy + conscientiousness * 
##     privacy + emotionalstability + openness, data = data2)
## 
## Residuals:
##     Min      1Q  Median      3Q     Max 
## -35.534  -8.500  -0.353   8.005  31.862 
## 
## Coefficients:
##                            Estimate Std. Error t value Pr(>|t|)    
## (Intercept)                71.46764   25.56331   2.796 0.005556 ** 
## gendermale                 -0.82720    1.54305  -0.536 0.592348    
## age                        -0.35049    0.13622  -2.573 0.010624 *  
## rolesstaff                 -2.86702    2.72803  -1.051 0.294236    
## rolesstudent              -13.24934    3.41818  -3.876 0.000134 ***
## familiarity                 0.09831    0.04321   2.275 0.023696 *  
## privacy                     0.11305    0.28545   0.396 0.692392    
## extraversion               -1.30956    0.65205  -2.008 0.045616 *  
## agreeableness             -16.11347    5.04150  -3.196 0.001561 ** 
## conscientiousness          15.43123    5.40578   2.855 0.004649 ** 
## emotionalstability          1.63395    0.78508   2.081 0.038366 *  
## openness                   -0.72602    0.82148  -0.884 0.377608    
## privacy:agreeableness       0.16950    0.05579   3.038 0.002615 ** 
## privacy:conscientiousness  -0.15879    0.06174  -2.572 0.010657 *  
## ---
## Signif. codes:  0 '***' 0.001 '**' 0.01 '*' 0.05 '.' 0.1 ' ' 1
## 
## Residual standard error: 12.21 on 266 degrees of freedom
## Multiple R-squared:  0.201,  Adjusted R-squared:  0.162 
## F-statistic: 5.148 on 13 and 266 DF,  p-value: 3.629e-08
```

```
lm.beta(model3b)
```

```
## 
## Call:
## lm(formula = score ~ gender + age + roles + familiarity + privacy + 
##     extraversion + agreeableness * privacy + conscientiousness * 
##     privacy + emotionalstability + openness, data = data2)
## 
## Standardized Coefficients::
##               (Intercept)                gendermale                       age 
##                0.00000000               -0.03102441               -0.26926466 
##                rolesstaff              rolesstudent               familiarity 
##               -0.08373501               -0.45904107                0.13701201 
##                   privacy              extraversion             agreeableness 
##                0.12365891               -0.11569687               -1.22247151 
##         conscientiousness        emotionalstability                  openness 
##                1.20980565                0.14671901               -0.05840817 
##     privacy:agreeableness privacy:conscientiousness 
##                1.56058559               -1.42143517
```

## Model Comparison

### Model 1 vs Model 2

```
anova(model1b, model2b)
```

```
## Analysis of Variance Table
## 
## Model 1: score ~ gender + age + roles + familiarity + privacy
## Model 2: score ~ gender + age + roles + familiarity + privacy + extraversion + 
##     agreeableness + conscientiousness + emotionalstability + 
##     openness
##   Res.Df   RSS Df Sum of Sq     F  Pr(>F)  
## 1    273 43422                             
## 2    268 41197  5    2225.1 2.895 0.01453 *
## ---
## Signif. codes:  0 '***' 0.001 '**' 0.01 '*' 0.05 '.' 0.1 ' ' 1
```

```
anova(model1b, model2b, test="Chisq")
```

```
## Analysis of Variance Table
## 
## Model 1: score ~ gender + age + roles + familiarity + privacy
## Model 2: score ~ gender + age + roles + familiarity + privacy + extraversion + 
##     agreeableness + conscientiousness + emotionalstability + 
##     openness
##   Res.Df   RSS Df Sum of Sq Pr(>Chi)  
## 1    273 43422                        
## 2    268 41197  5    2225.1  0.01286 *
## ---
## Signif. codes:  0 '***' 0.001 '**' 0.01 '*' 0.05 '.' 0.1 ' ' 1
```

### Model 2 vs Model 3

```
anova(model2b, model3b)
```

```
## Analysis of Variance Table
## 
## Model 1: score ~ gender + age + roles + familiarity + privacy + extraversion + 
##     agreeableness + conscientiousness + emotionalstability + 
##     openness
## Model 2: score ~ gender + age + roles + familiarity + privacy + extraversion + 
##     agreeableness * privacy + conscientiousness * privacy + emotionalstability + 
##     openness
##   Res.Df   RSS Df Sum of Sq      F   Pr(>F)   
## 1    268 41197                                
## 2    266 39662  2    1535.1 5.1477 0.006405 **
## ---
## Signif. codes:  0 '***' 0.001 '**' 0.01 '*' 0.05 '.' 0.1 ' ' 1
```

```
anova(model2b, model3b, test="Chisq")
```

```
## Analysis of Variance Table
## 
## Model 1: score ~ gender + age + roles + familiarity + privacy + extraversion + 
##     agreeableness + conscientiousness + emotionalstability + 
##     openness
## Model 2: score ~ gender + age + roles + familiarity + privacy + extraversion + 
##     agreeableness * privacy + conscientiousness * privacy + emotionalstability + 
##     openness
##   Res.Df   RSS Df Sum of Sq Pr(>Chi)   
## 1    268 41197                         
## 2    266 39662  2    1535.1 0.005813 **
## ---
## Signif. codes:  0 '***' 0.001 '**' 0.01 '*' 0.05 '.' 0.1 ' ' 1
```

## Regression Visualization

### Marginal Effects Plot

```
plot1 <- plot_model(model3b, type = "pred", terms = c("privacy", "agreeableness[1,4,7]"), title = "Marginal Effects", axis.title = c("Privacy Concerns", "Predicted Security Awareness Score"), legend.title = "Agreeableness")
plot2 <- plot_model(model3b, type = "pred", terms = c("privacy", "conscientiousness[1,4,7]"), title = "Marginal Effects", axis.title = c("Privacy Concerns", "Predicted Security Awareness Score"), legend.title = "Conscientiousness")
plot1
```

```
plot2
```

```
grid.arrange(plot1, plot2, ncol=2)
```
