# Supplementary figures and images for: Security awareness of single sign-on account in the academic community: the roles of demographics, privacy concerns, and Big-Five personality
Source: PeerJ Comput Sci. 2022 Mar 11;8:e918. doi: 10.7717/peerj-cs.918 (PMC9044249; doi:10.7717/peerj-cs.918)

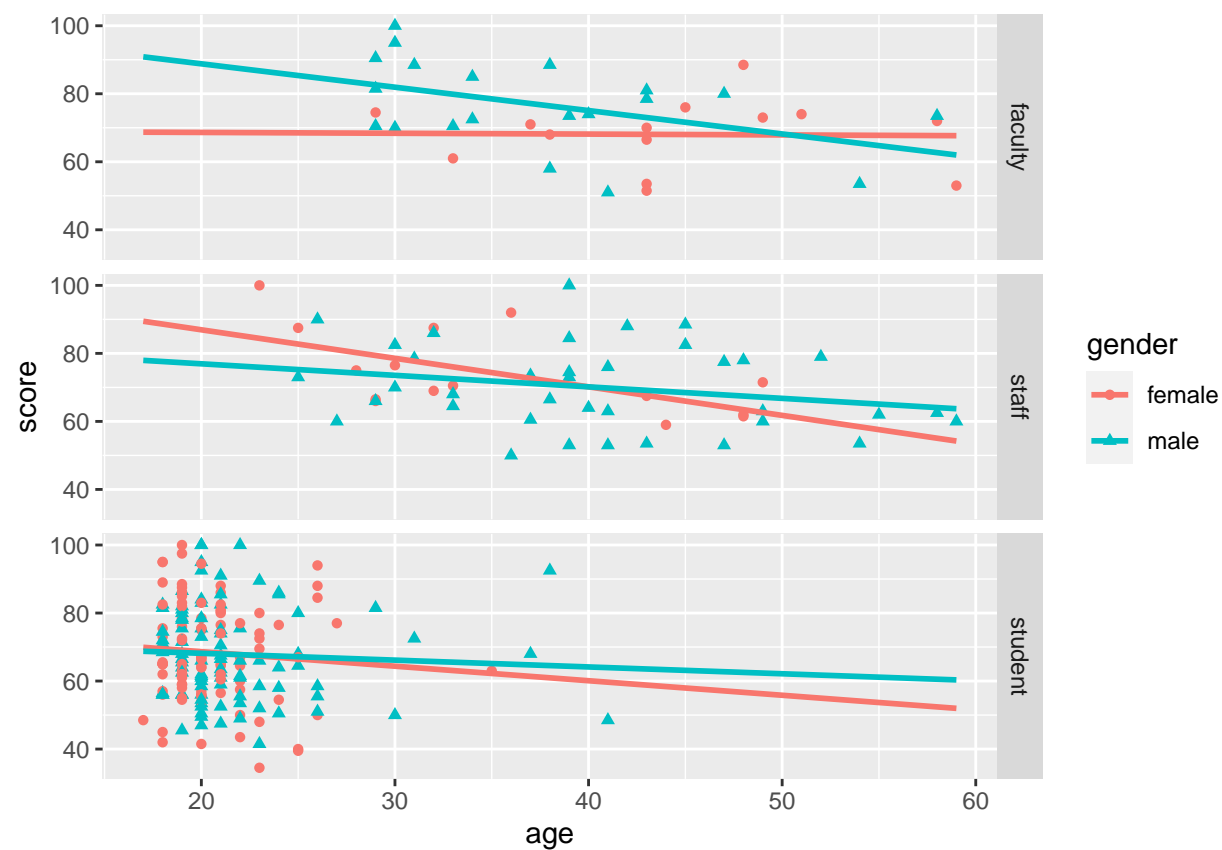

Supplement: Supplemental Information 1 [file peerj-cs-08-918-s001.zip › SSOSurveyStudy_files/figure-latex/unnamed-chunk-7-1.pdf]

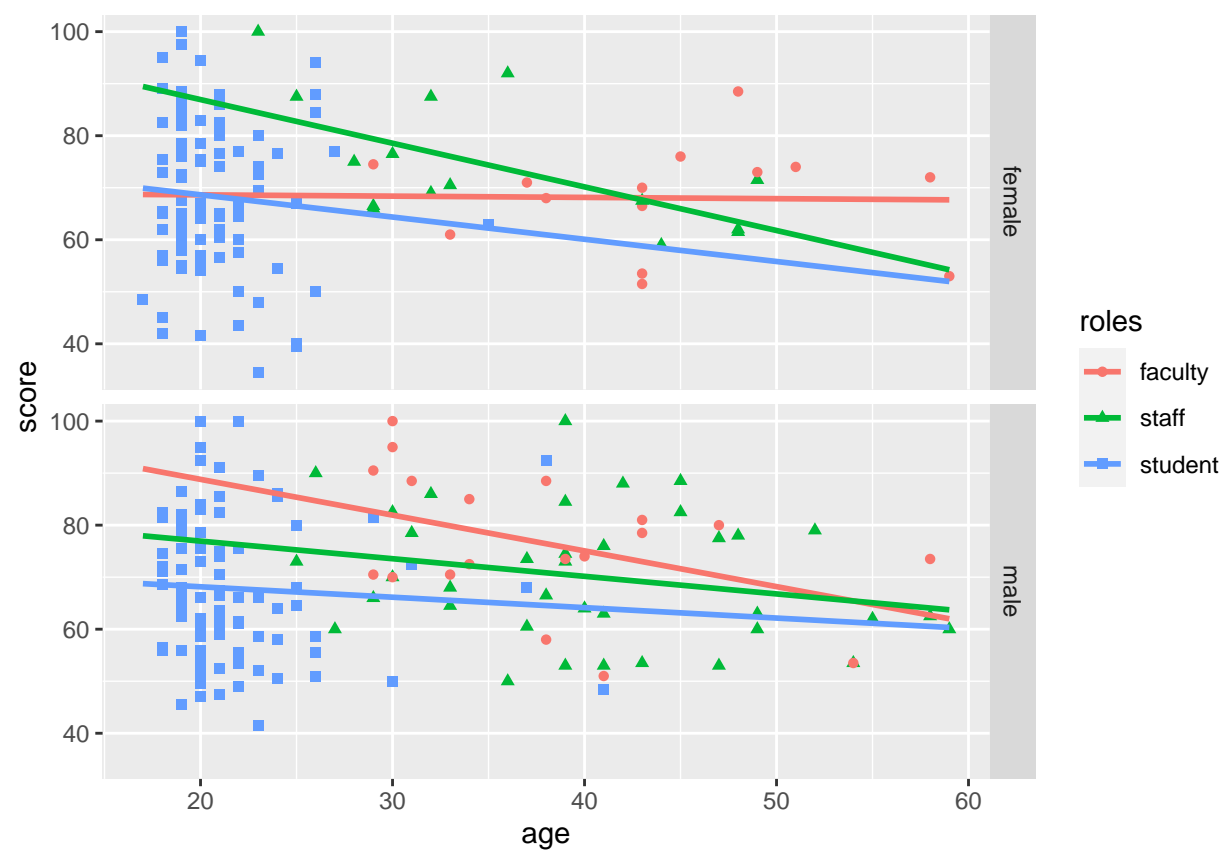

Supplement: Supplemental Information 1 [file peerj-cs-08-918-s001.zip › SSOSurveyStudy_files/figure-latex/unnamed-chunk-7-2.pdf]

# SSO Security Awareness by Age

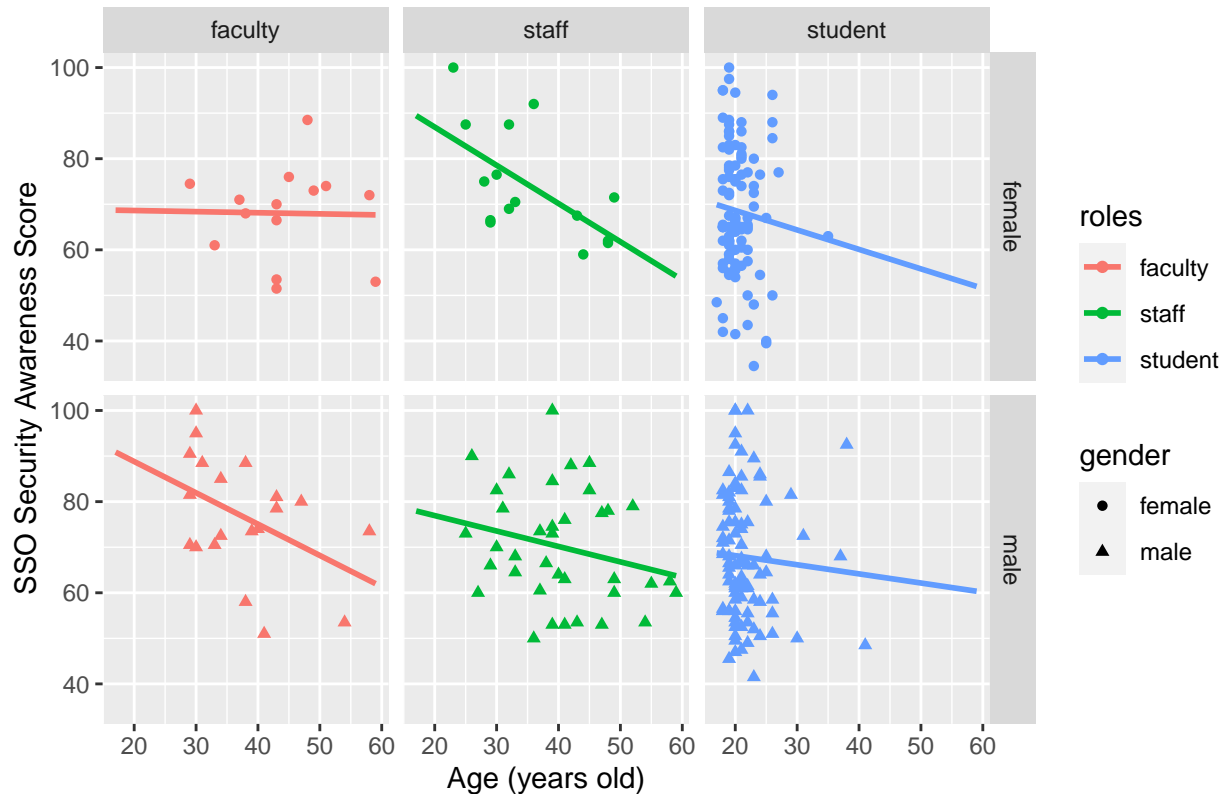

Supplement: Supplemental Information 1 [file peerj-cs-08-918-s001.zip › SSOSurveyStudy_files/figure-latex/unnamed-chunk-7-3.pdf]

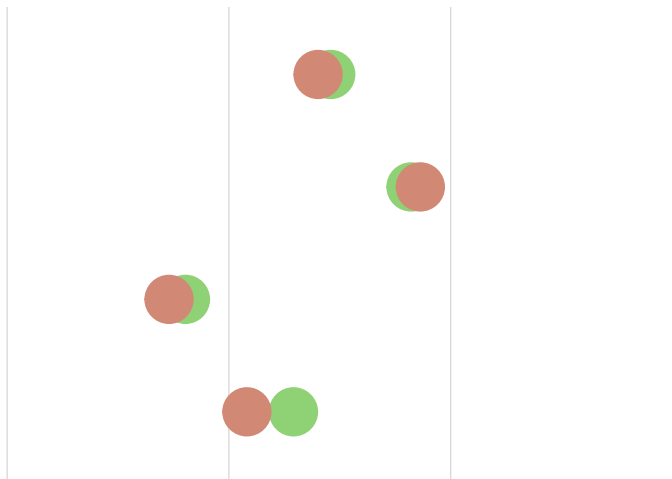

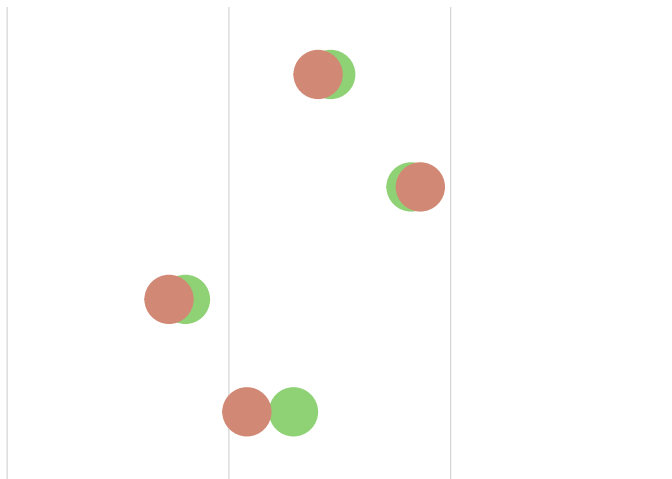

Supplement: Supplemental Information 1 [file peerj-cs-08-918-s001.zip › SSOSurveyStudy_files/figure-latex/unnamed-chunk-9-1.pdf]
