# Supplementary material for: Security awareness of single sign-on account in the academic community: the roles of demographics, privacy concerns, and Big-Five personality
Source: PeerJ Comput Sci. 2022 Mar 11;8:e918. doi: 10.7717/peerj-cs.918 (PMC9044249; doi:10.7717/peerj-cs.918)
Supplement: Supplemental Information 2 [file peerj-cs-08-918-s002.docx]

**Original measurement items in Bahasa Indonesia**

## SSO Familiarity

1. Saya tahu apa itu akun single sign on (SSO).
2. Saya tahu sistem dan data apa saja yang dapat diakses menggunakan akun SSO saya.
3. Saya sadar akan risiko dampak negatif apa saja yang dapat terjadi jika akun SSO saya digunakan oleh orang lain.

## Privacy Concerns

1. Secara umum, seberapa khawatir Anda akan privasi Anda ketika menggunakan internet?
2. Apakah Anda khawatir akan suatu organisasi yang identitas aslinya di dunia nyata tidak sama dengan pengakuannya di internet?
3. Apakah Anda khawatir akan orang yang pengakuannya di internet tidak sama dengan identitas aslinya di dunia nyata?
4. Apakah Anda khawatir akan pencurian identitas secara daring?
5. Apakah Anda khawatir pesan elektronik yang Anda kirimkan dapat dibaca oleh orang lain selain orang yang Anda tuju?

## Knowledge

1. Tidak ada larangan untuk menggunakan password yang sama untuk akun SSO dan akun pribadi seperti media sosial.
2. Tidak ada larangan untuk membagikan password akun SSO ke orang lain, termasuk teman atau kolega.
3. Kombinasi huruf besar, huruf kecil, angka, dan karakter khusus adalah sebuah keharusan saat memilih password, termasuk untuk akun SSO.
4. Tidak ada larangan untuk menggunakan password sepanjang 8 karakter atau kurang, termasuk untuk akun SSO.
5. Ketika login ke akun SSO di perangkat yang bukan milik sendiri, penggunaan mode incognito atau mode privat di web browser adalah sebuah keharusan.

## Attitude

1. Aman-aman saja menggunakan password yang sama untuk akun SSO dan akun pribadi seperti media sosial.
2. Membagikan password akun SSO ke orang lain, termasuk teman atau kolega, adalah ide buruk.
3. Aman-aman saja menggunakan password yang terdiri dari hanya kombinasi huruf saja, termasuk untuk akun SSO.
4. Password dengan panjang 8 karakter atau kurang itu sudah cukup aman, termasuk untuk akun SSO.
5. Login ke akun SSO di perangkat yang bukan milik sendiri tanpa mode incognito atau mode privat di web browser adalah suatu tindakan berisiko.

## Behavior

1. Saya menggunakan password yang berbeda untuk akun SSO dan akun pribadi seperti media sosial.
2. Saya berbagi password akun SSO dengan teman atau kolega.
3. Saya menggunakan kombinasi huruf besar, huruf kecil, angka, dan karakter khusus untuk semua password, termasuk akun SSO.
4. Saya selalu menggunakan password yang panjangnya lebih dari 8 karakter, termasuk untuk akun SSO.
5. Saya jarang menggunakan mode incognito atau mode privat di web browser ketika login ke akun SSO di perangkat yang bukan milik sendiri.
